# Embracing the Multifaceted Roles of Biomolecules in Biology and Medicine

**DOI:** 10.3390/biom15121636

**Published:** 2025-11-21

**Authors:** Gundu H. R. Rao

**Affiliations:** Laboratory Medicine and Pathology, Thrombosis Research, Lillehei Heart Institute, University of Minnesota, Minneapolis, MN 55455, USA; gundurao9@gmail.com

In recent peer-review discussions, a recurring critique has emerged: certain manuscripts, though scientifically rigorous, may fall outside the perceived thematic boundaries of the *Biomolecules*. As senior editor and a member of the advisory board, I see this as an opportune moment to reframe and reaffirm our editorial vision. Biomolecules, by their very nature, do not operate in silos [1]; rather, their identities and functions span a continuum of biological organization—from atoms to organisms, from biochemical reactions to systems-level physiology and pathology [2,3]. This continuum unfolds across multiple layers: The molecular level involves structure–function relationships, binding affinities, catalytic properties; the cellular level involves signaling pathways, regulatory loops, metabolic fluxes; the tissue and organ levels involve organ-specific functions, interactions, pathophysiological states; and the systemic and integrative levels involve whole-body responses, aging, disease progression, therapeutic outcomes.

Traditionally, the term “biomolecule” evokes DNA, RNA, proteins, lipids, and metabolites. Yet their significance extends beyond their chemical identities. They are the language of life—encoding, transmitting, and executing biological information [4]. They act as orchestrators of cellular dialogue, catalysts of adaptation, and sentinels of health and disease [5,6]. Their roles are dynamic and context-dependent: at times narrowly specialized, such as cleaving a substrate, and at other times serving as global regulators of development, immunity, metabolism, or cognition. Biomolecules operate within intricate networks—signal transduction, gene regulation, metabolic pathways—where interactions generate emergent behaviors. Systems biology integrates multi-omics datasets—genomics, proteomics, metabolomics—into computational models that reveal how complex cellular properties arise from molecular interactions. For example, biomolecular condensates, membrane-less assemblies that organize signaling and gene expression, have emerged as central regulatory hubs whose dysfunction is linked to disease [7].

At the molecular level, covalent and non-covalent interactions shape the three-dimensional architecture of proteins, nucleic acids, lipids, and carbohydrates [8]. Enzymes recognize substrates with exquisite specificity, reducing activation energy and accelerating biochemical reactions [9]. Molecular recognition—such as ligand binding to proteins or nucleic acids—is finely tuned and essential for biological function [10]. These molecular processes naturally scale to the cellular level, where ligand–receptor interactions initiate cascades that convert external signals into functional responses [11]. Platelet activation provides a vivid example: agonists such as thromboxane, ADP, collagen, thrombin, and epinephrine bind their receptors, triggering GTP-binding proteins, phospholipase C activation, and generation of second messengers (IP3 and DAG). IP3 mobilizes calcium from intracellular stores, while DAG activates protein kinase C—together driving platelet activation (Figure 1A,B). Antagonists such as adenosine, prostaglandin E_1_, prostacyclin (PGI_2_), and nitric oxide counteract this by lowering cytosolic calcium via adenylyl and guanylyl cyclases [12,13,14]. This illustrates a broader principle: biomolecules rarely act alone, and are instead elements of finely tuned regulatory networks.

Biomolecules also underpin metabolic regulation. They serve as building blocks, energy sources, and signaling mediators to maintain homeostasis [15]. Glucagon-like peptide-1 (GLP-1), for instance, is central to metabolic balance and the basis for advanced diabetes and obesity treatments. More broadly, tools such as flux-balance analysis and kinetic modeling reveal how reaction rates adapt to environmental changes [16]. At the tissue and organ levels, protein folding ensures function, while misfolding contributes to pathology [17]. Aging, with its hallmarks of genomic instability, telomere shortening, mitochondrial dysfunction, and chronic inflammation, highlights the systemic consequences of biomolecular dysfunction [18,19,20]. For example, liver cell senescence accelerates whole-body aging [19], while chronic inflammation contributes to diabetes, metabolic disorders, and neurodegeneration [20]. Classic case studies underscore these principles: hemoglobin’s oxygen binding demonstrates how subtle conformational shifts alter affinity, enabling efficient oxygen transport [21]. Kinases such as PI3K and MAPK regulate downstream pathways through precise catalytic mechanisms [22]. CRISPR-Cas9, an RNA-guided nuclease, exemplifies biomolecules as precision molecular tools now central to gene editing [23].

At the cellular scale, insulin binding initiates a cascade involving IRS-1, Akt, and GLUT4, regulating glucose uptake in adipocytes and muscle cells [24]. The tumor suppressor p53 senses DNA damage and determines whether to initiate repair, cell-cycle arrest, or apoptosis, depending on context [25]. Cytokines like IL-6 exemplify how biomolecules mediate immune communication, with effects determined by receptor distribution and co-signals [26]. At the organ level, cardiac troponins regulate heart muscle contraction and serve as biomarkers for myocardial infarction [27]. Apolipoprotein E isoforms influence lipid metabolism in the liver and brain, linking cardiovascular health to neurodegeneration [28]. In the kidney, renin and angiotensinogen collaborate within the RAAS to regulate blood pressure. At the systemic level, biomolecules orchestrate whole-body physiology and therapeutic outcomes. Leptin communicates adipose status to the hypothalamus, integrating metabolism across tissues. Cortisol coordinates systemic stress responses, influencing immunity, metabolism, and mood [29]. Therapeutically, monoclonal antibodies such as trastuzumab target specific biomolecules, altering cancer progression and survival [30]. Importantly, biomolecules are not static. Their roles shift with context: pro-inflammatory in one tissue, anti-inflammatory in another; beneficial during development, yet potentially oncogenic later in life. This adaptability reflects both their power and their complexity.

With this perspective, the *Biomolecules* seeks to be inclusive—not limited to classical biochemistry but welcoming interdisciplinary studies in systems biology, synthetic biology, clinical research, pharmacology, and translational science. Manuscripts that traverse traditional boundaries—linking structure to function, molecule to phenotype, or discovery to application—are not only welcomed but essential to advancing the field.

In an era when biology and medicine increasingly operate with molecular precision, let us foster a platform that reflects the true versatility and impact of biomolecules.

## Figures and Tables

**Figure 1:**
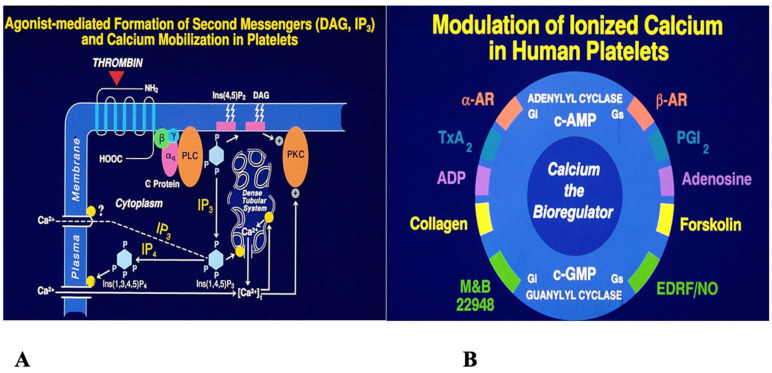
Signal transduction mechanisms involved in blood platelet activation. (Designed by the author and developed by artists at the University of Minnesota).

## Data Availability

Not applicable.
